# Patent Foods

**Published:** 1902-07-12

**Authors:** 


### Patent Foods.

So much money is spent in advertising patent foods of all sorts, kinds, and descriptions that it is hardly surprising that the question should sometimes arise in the mind of the purchaser how much is he paying for the actual food which he buys, and how much for being persuaded to buy it?

A little time ago a very interesting address on the subject was delivered before the South-West London Medical Society, by Dr. Robert Hutchison,[1] in which he expressed a very strong opinion as to the uselessness of many patent foods, and especially as to the monetary extravagance involved in their employment. He makes very light of the claims for greater digestibility which is set up on behalf of so many of them, and points out that there is a great deal of misconception abroad in regard to the necessity of peptonising or predigesting food. It is, however, when he comes to consider the price paid for these patent foods that he is most severe, for he sums the matter up by the general statement that not one of them is worth the money asked for it, and that some of them contain a ridiculously small amount of nourishment at the price. "Compare," he says, "the units of energy obtained by spending one shilling on some of the patent foods which are presented to you. In Valentine's Meat Juice you get six units of energy for a shilling. In Denaeyer's Peptone you get nine units of energy for a shilling. In Somatose, which is so largely advertised, there are 16½ units of energy for the same money. Now let us look at the amount of energy which we can get from certain natural foods. In meat we get 511 units of energy for a shilling, in egg 1,065 units, in milk 3,440 units, and in sugar nearly 5,000 units. So you are able to see from that how badly patent foods compare with ordinary foods in point of cost."

This statement is so strong that one feels instinctively and at once that there must be another side to the question. We must look at the evidence of those who consume these foods as well as at that of the chemist who analyses them. Take for example the above-mentioned meat juice. It is often given, of course, in fevers and to helpless people whose opinion on the subject is perhaps worthless. But it is often used by comparatively healthy and perfectly reasonable people who take it when they feel faint or exhausted, or done up with overwork. These people—whose evidence on any subject within their own ken would be accepted without hesitation—tell one that the stuff does them good and when one finds them flying on occasion to the same resource, year after year, one feels assured that in some way, although its utility as a food may be infinitesimal, it fulfils a mission of its own it gives these people something which they want. Indeed these so-called "foods" are in the same boat with beef tea, a preparation which, small as may be the number of units of energy which it may liberate per shilling of cost, still remains one of the most popular items of sick diet. If a patient fail to digest meat, even a dear extract may be worth the money.

The same is true in reference to some of the peptonised, malted, and cooked foods which are now so largely used. That they are not worth the money in units per shilling may be readily admitted; and that would be germane to the subject if the invalid could equally readily extract these units from all kinds of food. But when a dyspeptic, who has suffered agonies after "bread and milk," or has been blown up with flatulence after ordinary gruel, finds that he can quite comfortably take a cup of Benger or other similar compound, it is not by shillings but by pounds that he says their value is to be estimated.

No one can have much to do with dyspeptics of the more intelligent class without becoming aware how very differently the stomach responds to foods which apparently differ very slightly one from another. It is possible that the factor of importance often lies not in the food that is digested but in the residuum which is left; but in any case it may be pretty confidently stated that knowledge has not yet arrived at such a degree of accuracy as to enable us safely to reckon the value of a food by the number of units of energy it contains.

Hanging over the whole subject of patent foods, again, is the difficulty of the cook. If all sick people had good cooks how much greater might be the proportion of recoveries! The popularity and, we may add, the utility of many of the patent foods which flood the market lie in the ease with which a reasonably digestible, although often none too palatable form of nutriment can be prepared.

It may be true, and we believe it is true, that "an intelligent manipulation of flour, oatmeal, and infusion of malt" would render unnecessary many of the patent foods; but who is to manipulate? When manufacturers provide a food which can be prepared by aid of a little hot water or milk, they supply a thing that is wanted by thousands of sick people. The nutritive value is a detail. Shillings will not buy cooks.

[1] Reported in the Lancet, July 5.

